# Supplementary material for: Sustained-input switches for transcription factors and microRNAs are central building blocks of eukaryotic gene circuits
Source: Genome Biol. 2013 Aug 23;14(8):R85. doi: 10.1186/gb-2013-14-8-r85 (PMC4054853; doi:10.1186/gb-2013-14-8-r85)
Supplement: Additional file 5 — HTML Browsable Motif Output. Zipped folder containing all WaRSwap and FANMOD motif output, viewable in a web browser. [file gb-2013-14-8-r85-S5.ZIP › HTML_browsable_motif_output/WaRSwap_all_tair9/sigs_WSWRSLDraw-2000_all.pvals.heatmaps.html/motif_id_14_000101011_tftype_all_upstream_-2000_0.html]

```
BG_MODEL = Rnew2
MOTIF_ID = 14_000101011
TF_TYPE = all
UPSTREAM = -2000_0


PVals
FN_0.2	FN_0.4	FN_0.6	FN_0.8
dg_60.genes	0.403	0.003	0.017	0.683
dg_70.genes	0.319	0.001	0.019	0.659
dg_80.genes	0.772	0.08	0.192	0.553

ZScores
FN_0.2	FN_0.4	FN_0.6	FN_0.8
dg_60.genes	0.212	3.452	2.829	-0.846
dg_70.genes	0.401	3.794	2.884	-0.818
dg_80.genes	-0.822	1.586	0.648	-0.71

StDevs
FN_0.2	FN_0.4	FN_0.6	FN_0.8
dg_60.genes	199.924	177.884	73.982	14.582
dg_70.genes	196.7	170.061	75.066	13.845
dg_80.genes	153.59	138.603	61.793	11.822
```
